# Optimizing mean arterial pressure in septic shock: a critical reappraisal of the literature

**DOI:** 10.1186/s13054-015-0794-z

**Published:** 2015-03-10

**Authors:** Marc Leone, Pierre Asfar, Peter Radermacher, Jean-Louis Vincent, Claude Martin

**Affiliations:** Service d’Anesthésie et de Réanimation, Chemin des Bourrely, Hôpital Nord, Assistance Publique-Hôpitaux de Marseille, Aix Marseille Université, 13015 Marseille, France; Département de Réanimation Médicale et de Médecine Hyperbare Centre Hospitalier Universitaire Angers; and Laboratoire de Biologie Neurovasculaire et Mitochondriale Intégrée, CNRS UMR 6214 - INSERM U1083, Université Angers, PRES L’UNAM, 4 Rue Larrey, 49100 Angers, France; Institut für Anästhesiologische Pathophysiologie und Verfahrensentwicklung, Universitätsklinikum, Albert-Einstein-Allee 23, 89081 Ulm, Germany; Department of Intensive Care, Erasme Hospital, Université Libre de Bruxelles, route de Lennik 808, 1070 Brussels, Belgium

## Abstract

Guidelines recommend that a mean arterial pressure (MAP) value greater than 65 mm Hg should be the initial blood pressure target in septic shock, but what evidence is there to support this statement? We searched Pubmed and Google Scholar by using the key words ‘arterial pressure’, ‘septic shock’, and ‘norepinephrine’ and retrieved human studies published between 1 January 2000 and 31 July 2014. We identified seven comparative studies: two randomized clinical trials and five observational studies. The results of the literature review suggest that a MAP target of 65 mm Hg is usually sufficient in patients with septic shock. However, a MAP of around 75 to 85 mm Hg may reduce the development of acute kidney injury in patients with chronic arterial hypertension. Because of the high prevalence of chronic arterial hypertension in patients who develop septic shock, this finding is of considerable importance. Future studies should assess interactions between time, fluid volumes administered, and doses of vasopressors.

## Introduction

The blood pressure value that should be targeted during the management of septic shock is an important clinical issue. The mean arterial pressure (MAP) is one of the first variables that is monitored in these patients, and manipulation with vasopressor agents is relatively easy. Prolonged hypotension, defined as a MAP of less than 60 to 65 mm Hg, is associated with poor outcome [1,2].

The Surviving Sepsis Guidelines recommend that vasopressor therapy initially target a MAP of 65 mm Hg (grade 1C recommendation) [3] but that the actual value be individualized because the optimal MAP may be higher in older patients with atherosclerosis or previous hypertension (or both) than in younger patients without cardiovascular comorbidity [3]. After the initial resuscitation period, the ideal MAP target remains a matter of debate. In addition, a randomized clinical trial that tested the effects of norepinephrine versus a nitric oxide inhibitor in patients with septic shock suggested that an abrupt and sustained increase in MAP could be detrimental and was associated with an increased mortality rate [4]. However, it remains unclear whether the increased mortality was due to the adverse effect of the drug itself or to the excessive level of arterial pressure *per se*. We reviewed the published data related to MAP targets in septic shock and discuss the clinical implications.

## Overview of relationship between arterial pressure and organ perfusion

**Figure 1:**
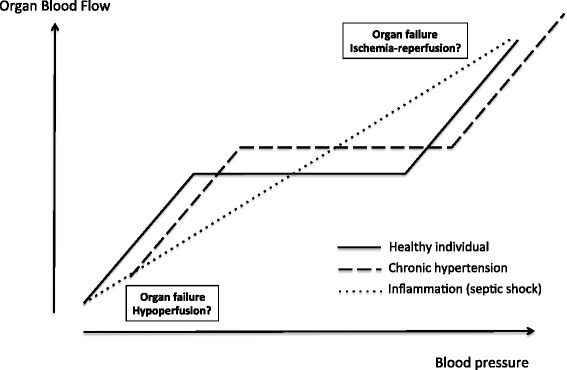
**Organ blood flow and blood pressure relationships in healthy individuals, individuals with chronic hypertension, and patients with septic shock.** The third linear relationship is theoretical.

Septic shock is characterized by both vasodilation and cardiac dysfunction, leading to a decrease in blood pressure. Hypotension generates organ failure due to hypoperfusion, with the MAP reflecting the driving pressure at the organ level. The goal of resuscitation is to restore adequate organ perfusion (that is, to optimize the relationship between oxygen needs and oxygen supply). In healthy individuals, blood flow remains constant over a large range of blood pressures, at least in the brain and the kidney. For many years, researchers have hypothesized that this autoregulatory mechanism is impaired in septic shock [5-7], so that increasing blood pressure will increase organ blood flow (Figure 1). In addition, the autoregulation threshold is dependent on the basal level of blood pressure, tending to be higher in patients with than in those without a prior history of arterial hypertension (Figure 1) [8]. The ‘optimal’ MAP target, therefore, may differ according to the patient’s medical history. Moreover, there are many autoregulatory thresholds depending on the specific tissue. In general, our goal is to provide an adequate perfusion to vital organs, which tend to have higher thresholds than less critical organs, such as skeletal muscle [9].

The microcirculation is markedly altered in septic shock [10,11]. Altered circulating cells, disseminated intravascular coagulation, peripheral edema, and impaired mitochondrial function have been implicated [12]. Interestingly, the microcirculation can still be impaired when the blood pressure goals of resuscitation, as recommended by guidelines [3], have been reached [13,14], suggesting that these hemodynamic goals may be inadequate for some patients (that is, not high enough) and that increasing blood pressure to higher levels may be warranted in some patients. On the other hand, these observations may imply that the microcirculation is at least partially independent of systemic hemodynamics, so that a further increase in arterial pressure may not improve microcirculatory blood flow or may even threaten it.

These concepts are based on the theoretically linear relationship between pressure and flow in septic states (Figure 1). To reach a high blood pressure target value, supplemental fluid volume and increasing dosages of inotropes or vasopressors or both are required. These drugs have adverse effects that are associated with organ failure [15], particularly true for catecholamines, the most common drugs used to maintain or increase blood pressure.

Experimentally, increasing arterial pressure excessively has harmful effects. In an ovine model of septic shock, Corrêa and colleagues [16] showed that targeting a MAP of between 50 and 60 mm Hg was associated with an increased incidence of acute kidney injury (AKI). In critically ill patients, unfavorable outcomes have been reported with persistent MAP values of less than 60 to 65 mm Hg [1,2]. Also, although MAP levels of 54 mm Hg have been associated with worse microcirculatory variables, increasing MAP to more than 65 mm Hg was associated with improved microcirculatory parameters [17]. Overall, data suggest that a level of MAP at 65 mm Hg is probably the minimal goal that should be targeted in septic shock. However, targeting a higher MAP (75 to 85 mm Hg) during resuscitation of otherwise healthy swine with fecal peritonitis-induced septic shock did not ameliorate the inflammatory response and resulted in increased net positive fluid balance and vasopressor load during resuscitation [16]. Hence, a MAP target that is too low may be associated with organ hypoperfusion, whereas one that is too high may be associated with ischemic injury due to excessive vasoconstriction.

## Literature review

### Methods

Pubmed and Google Scholar were searched by using the key words ‘arterial pressure’, ‘septic shock’, and ‘norepinephrine’. The search was limited to studies published between 1 January 2000 and 31 July 2014. Only randomized clinical trials, comparative studies, and observational studies were analyzed; reviews and editorials were excluded as were animal studies and studies published in languages other than English.

### Results

We identified 12 studies; seven were comparative studies determining the effect of different goals of blood pressure on outcome (Table 1) [18-24], and five were observational studies (Table 2) analyzing hemodynamic variables in patients with septic shock to determine the influence of arterial pressure on outcomes [1,2,25-27]. The end-points were mortality, regional circulation, and microcirculation in four [1,2,24,25], five [18-20,26,27], and three [21-23] studies, respectively. Among the seven comparative studies, six studies included fewer than 30 patients [18-23]; one of the two randomized clinical trials included 776 patients [24]. Because of the relative weight of this latter study, it will likely have a larger impact on future guidelines than the smaller studies. Nevertheless, the smaller studies collected useful additional informative data that were not included in the large study. Surprisingly, severity scores were similar in the different studies, although mortality rates were highly variable, ranging from 17% to 76%.

**Table 1:** **Studies comparing different levels of mean arterial pressure in septic shock**

**Reference**	**Study design**	**Number of patients**	**Targeted MAP, mm Hg**	**Primary outcomes**	**Severity score**	**28-day mortality, %**
LeDoux *et al.* [18] (2000)	Prospective cohort	10	65, 75, 85	Regional circulation and oxygen metabolism	APACHE II: 29	70
Bourgoin *et al.* [19] (2005)	Randomized clinical trial	28	65, 85	Regional circulation and oxygen metabolism	APACHE II: 27	NA
Deruddre *et al.* [20] (2007)	Prospective cohort	11	65, 75, 85	Renal perfusion	SAPS II: 57	NA
Jhanji *et al.* [21] (2009)	Prospective cohort	16	60, 70, 80, 90	Microcirculation	APACHE II: 23	62.5
Thooft *et al.* [22] (2011)	Prospective cohort	13	65, 75, 85, 65	Microcirculation	APACHE II: 23	17
Dubin *et al.* [23] (2009)	Prospective cohort	20	65, 75, 85	Microcirculation	APACHE II: 24	50
Asfar *et al.* [24] (2014)	Randomized clinical trial	776	65, 85	28-day mortality	SAPS II: 57	35

APACHE II, Acute Physiology and Chronic Health Evaluation II; MAP, mean arterial pressure; NA, not available; SAPS II, Simplified Acute Physiology Score II.

**Table 2:** **Observational studies assessing the effect of mean arterial pressure on outcomes**

**Reference**	**Type of study**	**Number of patients**	**Primary outcomes**	**MAP, mm Hg**	**Severity score**	**28-day mortality, %**
Varpula *et al.* [1] (2005)	Retrospective cohort	111	30-day mortality	65	APACHE II: 17	33.3
Dünser *et al.* [2] (2009)	Retrospective cohort	274	28-day mortality	>60	SAPS: 52 APACHE II: 27	27.7
Dünser *et al.* [25] (2009)	Post hoc analysis	290	28-day mortality	>70	SAPS II: 58	76
Badin *et al.* [26] (2011)	Prospective cohort	217	Acute kidney injury at 72 hours	72-82	SAPS II: 53	39^a^
Poukkanen *et al.* [27] (2013)	Prospective cohort	423	Acute kidney injury at day 5	73	SAPS II: 40	24^a^

^a^Hospital mortality was reported instead of 28-day mortality. APACHE II, Acute Physiology and Chronic Health Evaluation II; MAP, mean arterial pressure; NA, not available; SAPS II, Simplified Acute Physiology Score II.

#### Mean arterial pressure and macro-hemodynamics

MAP was increased from 65 to 85 mm Hg in six of the comparative studies [18-20,22-24] and to 60, 70, 80, and 90 mm Hg in one study [21]. Overall in the studies, the increase in MAP was achieved by a 1.7 ± 0.4 μg/kg per minute increase in norepinephrine infusion. Heart rate was not affected by the increase in norepinephrine infusion, but cardiac output increased. Because the increase in MAP was greater than the increase in cardiac output, systemic vascular resistance increased (Table 3). Mean pulmonary arterial pressure changed inconsistently.

**Table 3:** **Hemodynamic variables for mean arterial pressure targets of 65 and 85 mm Hg**

**Variables**	**References**	**Number of patients**	**65 mm Hg** ^**a**^	**85 mm Hg** ^**a**^
Heart rate, beats per min	[16-22]	874	100 ± 11	101 ± 11
Cardiac index, L/min per m^2^	[16-21]	98	3.9 ± 1.9	4.3 ± 1.2
SvO_2_ or ScvO_2_, %	[16,17,19-21]	87	74 ± 2	75 ± 3
Lactate, mmol/L	[16-21]	98	2.3 ± 0.3	2.2 ± 0.4
Norepinephrine, μg/kg per min	[16-21]	874	0.47 ± 0.38	0.79 ± 0.52

^a^With respect to reference [19], values were derived from the calculation of mean values between 60 and 70 mm Hg for 65 mm Hg and between 80 and 90 mm Hg for 85 mm Hg. MAP, mean arterial pressure; ScvO_2_, central venous oxygen saturation; SvO_2_, mixed venous oxygen saturation.

#### Effects on organ function

The effects on organ function of increasing MAP from 65 to 85 mm Hg were assessed in two comparative studies [18,19], which included a total of 38 patients. The first study included 10 patients in whom MAP was progressively increased to three levels (65, 75, and 85 mm Hg) [18]. In the second study, variables were measured at a MAP of 65 mm Hg and then the MAP was either kept at 65 mm Hg or increased to 85 mm Hg [19]. The findings were consistent in the two studies and showed no differences in gastric or renal circulations with the different MAP targets. In the three observational studies that assessed the effect of different MAPs on organ function, a MAP of less than 75 mm Hg was associated with development of AKI [1,26,27].

Deruddre and colleagues [20] prospectively studied the relationship between MAP and organ function, increasing the MAP from 65 to 75 and 85 mm Hg by increasing the norepinephrine infusion rate in 11 patients with septic shock. The renal resistive index measured by pulsed Doppler in the interlobar renal arteries decreased and urine output increased when MAP was increased from 65 to 75 mm Hg but not when MAP was increased from 75 to 85 mm Hg. Creatinine clearance was unaffected.

#### The effects of mean arterial pressure on oxygen metabolism

Oxygen exchange was measured in five of the comparative studies [18,19,21-23]. Increase in cardiac output was associated with an increase in oxygen delivery; whole body oxygen uptake did not change. Plasma lactate levels were unaffected. The effects of increasing MAP on mixed venous oxygen saturation or central venous oxygen saturation were inconsistent: mixed venous oxygen saturation/central venous oxygen saturation increased in three studies [18,20,21] and was unchanged in two studies [18,23]. This discrepancy may be due to several factors, including the preload of patients and their cardiac function. The magnitude of change in oxygen variables did not appear to be clinically relevant (Table 3).

#### Effects on the microcirculation

The effects of an increase in MAP on the microcirculation were variable. Using sidestream darkfield (SDF) imaging, Dubin and colleagues [23] increased MAP to 65, 75, and 85 mm Hg and measured sublingual capillary microvascular flow index or the proportion of perfused capillaries. In patients with septic shock, increasing MAP improved microcirculatory variables in the patients with impaired microcirculation at baseline. In contrast, the microcirculation was impaired when baseline conditions were normal. This underlines the need for an individualized assessment. Using near-infrared spectroscopy with vaso-occlusive tests, Thooft and colleagues [22] found that perfusion was slightly improved when MAP was increased from 75 to 85 mm Hg but that it was impaired when MAP was decreased to 65 mm Hg. However, these effects were quite variable from one patient to another. In a study using measurement of intra-cutaneous oxygen partial pressure via a Clark electrode coupled with laser Doppler flowmetry, Jhanji and colleagues [21] found that increase in MAP was accompanied by a significant increase in cutaneous oxygen partial pressure and red blood cell flow, whereas the sublingual microcirculation explored by SDF imaging was unaffected.

#### Effects on mortality

In two of the observational studies, MAP levels of less than 60 to 70 mm Hg were independent determinants of mortality [1,2]. However, in a *post hoc* analysis, Dünser and colleagues [25] found no association between MAP and mortality.

In a large randomized clinical trial of 776 patients, Asfar and colleagues [24] compared the effects of low-target and high-target MAP on mortality. The study was designed to show an absolute 10% difference in mortality at day 28, assuming a mortality rate of 45%. The patients were allocated to protocols targeting MAPs of 65 to 70 mm Hg or 80 to 85 mm Hg for 5 days. As expected, the doses of norepinephrine were higher in the high-MAP target group than in the low-target group, but the cumulative fluid balance was similar. The 28-day mortality rate was 35% in both groups. The only difference was a higher rate of *de novo* atrial fibrillation in the high-target group (6.7% versus 2.8%, *P* = 0.02). Chronic arterial hypertension was reported in 44% of patients, and in a predefined stratum analysis of these patients, targeting a MAP of 80 to 85 mm Hg was associated with better renal function (lower rate of serum creatinine doubling and renal replacement therapy requirements). However, as also reported in one of the observational studies [27], there was no clear relationship between mortality and AKI. Consequently, this finding would suggest that targeting a MAP of 65 to 70 mm Hg or 80 to 85 mm Hg has similar effects on survival in a heterogeneous population of patients with septic shock. Of note, one limitation of this study [24] was that the actual MAP achieved in the low-target group was 75 mm Hg instead of 65 to 70 mm Hg.

#### Norepinephrine dosages and levels of mean arterial pressure

In the comparative studies, the doses of norepinephrine were increased by about 63% in order to increase MAP from 65 to 85 mm Hg [18-24]. Hence, patient management *per se* may have impacted outcomes in that not only was the level of MAP altered but also the dosages of vasopressors. One of the observational studies analyzed the interactions between MAP, norepinephrine load, and outcome [25]. The norepinephrine load was associated with mortality with a relative risk of 1.83 and a 95% confidence interval of between 1.40 and 2.38. This load was also associated with several disease-related events, and these associations were independent of age, prior history of hypertension, and co-morbidity. This study, therefore, suggests that the norepinephrine load, rather than the MAP *per se*, was associated with impaired outcomes. This limitation, which is related to the design of these studies, makes it difficult to differentiate between the effects of blood pressure and those of norepinephrine.

## Clinical implications

MAP may be a relevant goal for improving outcomes in septic shock but, although in general a low-MAP target strategy seems to be similar to a high-target strategy in terms of outcomes [24], a single fixed value is not suitable for all patients. The optimal blood pressure target likely ranges from 65 to 85 mm Hg and probably lies between 65 and 75 mm Hg in most patients. High MAP targets are associated with adverse effects, including atrial fibrillation, probably due to high doses of vasopressors. In patients with chronic hypertension, a level closer to 85 mm Hg may be associated with less renal impairment [24]. In support of these observations, in their study of early goal-directed therapy, 6 hours after starting resuscitation, Rivers and colleagues [28] found that the MAPs were 95 mm Hg in the early-goal directed therapy group and 81 mm Hg in the control group. In this study, around 66% of the patients had chronic hypertension. As survival was increased in the early-goal directed therapy group, these findings support the targeting of a higher MAP in patients with chronic hypertension [28]. However, this finding was not confirmed in recent studies [29,30]. Finally, the ‘optimal’ MAP differs between patients and within the same patient over time. Hence, there is a need for repeated examination to confirm the adequacy of organ function at the chosen MAP.

A 1-day audit of intensive care practice showed that a MAP goal was pre-fixed in only 70% of patients with septic shock [31]. In most patients, physicians seem to target a MAP of greater than 65 mm Hg. However, there is still considerable uncertainty about the relationship between fluid resuscitation, vasopressors, and the timing of these hemodynamic interventions. An interesting retrospective study by Waechter and colleagues [32] assessed the interactions between the three variables. This study suggested that the focus during the first hour of resuscitation be aggressive fluid administration. The timing of vasopressor agents was also important, with the lowest mortality rates associated with starting vasoactive agents 1 to 6 hours after hypotension onset. The duration of hypotension (defined as a MAP of less than 60 mm Hg) was an important predictor of mortality [32]. Hypotension should, therefore, be corrected in all patients without delay.

**Figure 2:**
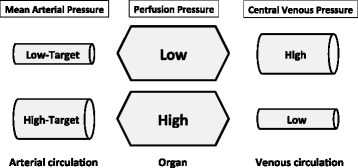
**Interactions between mean arterial pressure, central venous pressure, and perfusion pressure.**

Central venous pressure (CVP) is often inaccurate for predicting the need for volume expansion [33-35]. However, it represents the downstream pressure, whereas the perfusion pressure determinants are upstream and downstream pressures (Figure 2). The increase in downstream pressure generates congestion [36]. Thus, the optimal MAP most likely also depends on the CVP level. However, CVP does not always reflect the downstream pressure, because of the presence of Starling resistor phenomena in some vascular beds. The Surviving Sepsis Campaign recommends reaching a MAP of at least 65 mm Hg and at the same time a CVP of at least 12 mm Hg (in mechanically ventilated patients) [3]. However, in terms of organ perfusion, the optimal difference between MAP and CVP remains unclear.

It has been suggested that the microcirculation could be a critical end-point for resuscitation of septic shock. Microcirculatory assessment has been facilitated by the development of non-invasive devices, such as SDF imaging or near-infrared spectroscopy [37-39]. Several observational studies have shown that the microcirculation is markedly altered in patients with septic shock [12,13,40] and that patients with persisting microcirculatory impairment have poorer outcomes than other patients [17,40,41]. An improvement in the microcirculation is associated with increased survival [41], but it is not yet known whether improving microcirculatory blood flow by a pharmacologic intervention can improve outcome from septic shock. Increasing MAP had variable effects on the microcirculation in the four studies that reported this parameter [20-23]. Whether these discrepancies are related to the tool used or to patient differences or the site of measurement or to a combination of these remains unknown. Further studies are required to assess the relevance of these measurements and the type of intervention (that is, increase in flow or pressure or both). The ultimate and definitive end-point would be to demonstrate a relationship between change in microcirculatory blood flow and improvement in organ function and, at best, survival.

## Conclusions

The results of the SEPSISPAM (Sepsis and Mean Arterial Pressure) study [24] suggest that a MAP target of 65 to 75 mm Hg is usually sufficient in patients with septic shock, but a higher MAP (around 75 to 85 mm Hg) may be preferable in patients with chronic arterial hypertension. This issue is of major clinical importance in view of the high prevalence of chronic hypertension in patients admitted to intensive care units with septic shock and of the high morbidity associated with AKI. Recent guidelines recommend initially targeting a MAP level of more than 65 mm Hg and a higher MAP in septic patients with a history of hypertension who respond to a higher blood pressure [42].

Our results encourage the development of monitoring at the bedside to help determine the optimal level for each individual, although whether an individual-based approach will result in better outcomes than a protocol-based approach targeting a pre-determined level of MAP in all patients remains to be demonstrated [30]. Importantly, the delay to achieve the target is probably as important as the target itself. Finally, the technique used to increase MAP (amount of fluids, association of vasopressors) needs further investigation, in particular in patients with chronic arterial hypertension.

## Key messages

- In heterogeneous populations of patients with septic shock, there is no difference in survival rates for target mean arterial pressure levels of between 65 and 85 mm Hg.
- In patients with a history of arterial hypertension, a mean arterial pressure level of greater than 75 mm Hg may protect against progression to acute kidney injury.

## Abbreviations

AKI: Acute kidney injury
CVP: Central venous pressure
MAP: Mean arterial pressure
SDF: Sidestream darkfield
